# The Mediterranean diet and dietary approach to stop hypertension (DASH)-style diet are differently associated with lipid profile in a large sample of Iranian adults: a cross-sectional study of Shahedieh cohort

**DOI:** 10.1186/s12902-021-00856-w

**Published:** 2021-09-22

**Authors:** Monireh Panbehkar-Jouybari, Mehdi Mollahosseini, Amin Salehi-Abargouei, Hossein Fallahzadeh, Masoud Mirzaei, Mahdieh Hosseinzadeh

**Affiliations:** 1 Nutrition and Food Security Research Center, Shahid Sadoughi University of Medical Sciences, Yazd, Iran; 2 Department of Nutrition, School of Public Health, Shahid Sadoughi University of Medical Sciences, Medical Campus, Alem Sq, Yazd, Iran; 3 Research Center of Prevention and Epidemiology of Non-Communicable Diseases, Departments of Biostatistics and Epidemiology School of Public Health, Shahid Sadoughi University of Medical Sciences, Yazd, Iran; 4 Yazd Cardiovascular Research Centre, Shahid Sadoughi University of Medical Science, Yazd, Iran

**Keywords:** Cohort, Dietary pattern, Lipid profile, LDL, HDL, TC

## Abstract

**Background:**

The association between the Mediterranean diet (MED) or dietary approach to stop hypertension (DASH) and cardiovascular disease (CVD) risk factors is well-documented. Nevertheless, a consistent relationship with the Middle East population has yet to be known. Thus, we aimed to investigate the association between DASH/MED and blood lipids in Iranian adults.

**Methods:**

Four thousand seven hundred forty participants, aged 35–70 years (mean: 50.0) participated in the Shahedieh cohort study in Yazd, Iran, were followed from 2016 until now. Participants provided dietary and blood lipid data through a validated semi-quantitative food frequency questionnaire, and blood samples were taken after a fasted state. We used binary logistic regression to examine the association between DASH/MED scores and blood lipids.

**Results:**

In the participants who ingested a DASH-like diet the third vs. the first tertile of total cholesterol (TC), triglyceride (TG), low-density lipoprotein (LDL) levels, and LDL/HDL (high-density lipoprotein) ratio reduced significantly (*P* < 0.01). While in the participants who ingested the MED-like diet the HDL level increased significantly( 52.8 ± 12. 3 vs. 51.6 ± 11.6, *P* < 0.01). In Binary logistic regression, higher adherence to the DASH diet showed 19 % lower odds of high TC level (OR: 0.81; 95 %CI: 0.69–0.95) and 18 % lower odds of high LDL/HDL ratio (OR: 0.82; 95 %CI: 0.70–0.96). Besides, high adherence to the MED diet was associated with lower odds of LDL/HDL ratio (OR: 0.85; 95 %CI: 0.72–0.99).

**Conclusions:**

Our findings suggest that TC, TG, LDL, LDL/HDL ratio, and HDL improved in participants who ingested a DASH-like diet and the LDL/HDL ratio improved in participants who ingested MED-like diet and, subsequently they might have a protective effect on CVDs risk. Further epidemiological studies are needed to confirm our findings.

## Introduction

Cardiovascular disease (CVD) is the leading cause of mortality worldwide (31 % of global deaths) [1]. Considering the link between blood lipid metabolism and the development of atherosclerosis, lowering serum cholesterol concentration has been the primary strategy for the prevention and treatment of CVD [2, 3]. The National Cholesterol Education Program has recommended that therapeutic lifestyle change should be the primary treatment for lowering cholesterol values, and pharmacological treatment should be practiced in cases who don not response to lifestyle modification. Advocated modifications include dietary changes, increased physical activity, and weight management. The association between consumption of fish, fruits, vegetables, and whole grains with CVD is shown by several studies [4–6]. To improve LDL, dietary modifications include limiting saturated fat to < 7 % of calories and cholesterol to < 200 mg/d, increasing viscous fiber (10–24 g/d), and plant stanols/sterols (2 g/d) is recommended [3]. Previous studies have shown that the Mediterranean diet is associated with a lower CVD risk [7, 8].

The traditional Mediterranean diet (MED) focuses on a high intake of olive oil, fruits, nuts, vegetables, and grains; moderate intake of fish and poultry; low intake of dairy products, red meat, processed meat, and sweets; and moderate consumption of wine [9]. In observational cohort studies [10, 11] and a secondary prevention study (the Lyon Diet Heart Study) [12], increased adherence to the MED resulted in a positive effect on CVD’s risk [10–12] and can prevent coronary heart disease [13]. Moreover, the Dietary Approaches to Stop Hypertension (DASH) diet is rich in fruits, vegetables, and low-fat dairy products, contains grains, poultry, fish, and nuts and limits saturated fat, red meat, sweets, and sugar-containing beverages. The DASH diet contains lower amounts of total fat, saturated fat, and cholesterol while providing higher amounts of potassium, calcium, magnesium, fiber, and protein. Therefore, some studies have investigated other beneficial effects of this dietary pattern such as reducing insulin resistance and controlling fasting blood glucose and lipid profiles [14–16]; suggesting this dietary pattern, may also be a useful for the preventing the risk of CVDs and lipid profile.

Some studies, however, have found no link between the DASH/MED diet and lipid profile [17, 18]. To the best of our knowledge, there are a limited number of studies that investigated the association between these dietary patterns and lipid profiles in Middle East countries such as Iran and most of them had a limited sample size [17, 19–21]. We hypothesized that the DASH and MED patterns could have a positive effect on blood lipids with different lipid-modifying since food groups of the MED and DASH vary. Therefore, this study aimed to evaluate the association between DASH/MED and lipid profile in a large sample of Iranian adults living in central Iran.

## Materials and methods

### Study design and population

The present cross-sectional study was conducted on the data from recruitment phase of the Shahedieh cohort study which is part of the PERSIAN multicenter cohort study conducted on a representative sample of an adult Iranian population (age: 35–70 y). For the Shahedieh cohort study, 9971 adults living in three cities of Yazd Greater Area (Shahedieh, Zarch, and Ashkezar), located in Yazd Province, Iran, from year 2016 to present were recruited. Detailed information about the protocol of the PERSIAN cohort study is provided elsewhere [22]. In brief, the participants were selected by a multistage cluster random sampling method after providing written informed consent. In the case of illiterates, informed consent was obtained from their legally authorized representatives. Eligible participants were asked to provide blood samples and data on general characteristics, as well as demographic, dietary intake, smoking, and other lifestyle-related data via validated questionnaires. Anthropometric and blood pressure measurements were also performed on all participants. All data were collected by trained interviewers [22].

Data were provided from 9971 adults. Participants with CVDs including cardiac ischemia, myocardial infarction, stroke (*n* = 241), and different types of cancer (e.g., skin cancer, breast cancer, gastric cancer, colorectal cancer, and bladder cancer), and a history of hematopoietic cancers (*n* = 29) were excluded from the study because of the possibility of a dietary modification. Data from participants with a history of fatty liver (*n* = 511), thyroid disease (*n* = 609), diabetes (*n* = 1684), hypertension (*n* = 1183), gestational diabetes mellitus (GDM) (*n* = 178), preeclampsia (*n* = 80), and participants with body mass index (BMI) < 18.5 and BMI > 40 (*n* = 130) were also omitted. Furthermore, we excluded participants who left > 70 items in the food frequency questionnaire (FFQ) unanswered as well as participants with under/over-reporting (e.g., daily energy intake < 800 kcal/d or > 6500 kcal/d; *n* = 315). The missing data consisted of 271 participants who were also excluded from the study. After the aformentioned exclusions, 4740 participants remained for the present analysis. All the experimental protocols were conducted in accordance with the guidelines of the Declaration of Helsinki. Also, the present study was approved by the ethics committee of the Shahid Sadoughi University of Medical Sciences (IR.SSU.SPH.REC.1398.017).

### Dietary assessment

The validated semi-quantitative FFQ with 178 items was administered as an interview by trained interviewers to assess dietary foods and supplements [23]. The original semi‐quantitative FFQ contains 168 items, then 10 additional foods commonly consumed in Yazd were added. the study of zimorovat et al. [23] indicated that: Participants attended a large long-term clinical trial were asked to complete three semi-quantitative FFQs by interview, and nine 3-day weighed dietary records (WDRs), over 9 months. The ICC between FFQs for repeatability ranged from 0.43 (thiamin) to 0.73 (vitamin D, median: 0.56). The de-attenuated, age, sex, and education adjusted correlation coefficient between the last FFQ and weighted food frequency questionnaires (WDRs) ranged from − 0.05 for vitamin A to 0.41 for manganese (median: 0.26). The median exact agreement and complete disagreement between FFQ3 and WDRs were 33 and 6 %, respectively. The FFQ3 validity coefficient for vitamin C, calcium, magnesium, and zinc were 0.13, 0.62, 0.89, and 0.66, respectively, using the triads method. Study participants were asked to answer two questions about the frequency of food consumption (number of times per month, week, or day) for each of the 178 food in the past year, as well as the amount of food frequently consumed based on standard portion sizes commonly consumed by Iranians. All foods were converted to grams/day based on household portion size of food intakes [24]. The Iranian food composition table (FCT), and U.S. Department of Agriculture FCT for the foods that were not available in the Iranian FCT were used to calculate energy and nutrient intakes [25, 26].

### Adherence to MED diet

We used a modified scale constructed by Trichopoulou et al. to indicate the degree of adherence to the MED diet [27]. Each of the eight indicated components was assigned a value of 0 or 1, using the sex-specific median as the cutoff. For beneficial components (vegetables, legumes, fruits and nuts, grains, and fish), a value of 0 was considered for individuals if their consumption was below the median, and a value of 1 was considered for individuals whose consumption was at or above the median. For components that are presumed to be harmful such as meat, poultry, and dairy products, individuals with below the median’s consumption were assigned a value of 1, and individuals with at or above the median’s consumption were assigned a value of 0. Finally, for fat intake, the ratio of monounsaturated to saturated fats was used. Alcohol consumption was excluded from the scale because, there is no reliable data on alcohol consumption, in Iran. Thus, the total Mediterranean- diet score ranged from 0 (minimum adherence to Mediterranean diet) to 8 (maximum adherence).

### Adherence to the DASH diet

To examine the degree of adherence to the DASH diet, we constructed the DASH scores based on foods and nutrients emphasized or minimized in the DASH diet, focusing on eight components: high intake of fruits, vegetables, nuts and legumes, dairy products, and low intake of grains, sugar-sweetened beverages (SSB) and sweets, sodium, and red and processed meats [28]. Then, subjects were categorized into deciles of foods and nutrients. participants whose consumption was in the highest decile of total grains, fruits, vegetables, dairy products, nuts, and legumes were assigned a score of 10; and persons whose consumption was in the lowest decile were assigned a score of 1. Moreover, individuals with the highest consumption of red and processed meat, SSB and sweets, and sodium were assigned a value of 1; and those with the lowest consumption were assigned a value of 10. Finally, the scores of eight components were summed up to obtain the overall DASH score for each participant. The lowest and highest DASH scores ranged from 8 (minimal adherence) to 40 (maximal adherence), respectively.

### Laboratory assessment

Blood samples (25 mL) were collected from the participants after an overnight fasting state (8–12 h before blood sampling). The blood samples were aliquoted into serum, buffy coat, and whole blood samples. Serum total cholesterol (TC), low-density lipoprotein cholesterol (LDL), high-density lipoprotein cholesterol (HDL), and triglyceride (TG) were determined from the serum samples by an auto-analyzer (Analyzer BT1500) using Pars Azmun standard kits. For every batch kits, we compared with Normal control and Patient control kits. Also the auto-analyzer was calibrated regularly. The high/low lipid profile was defined based on the EAC/EAS guidelines as follows: Serum LDL ≥ 130 mg/dL for men and women; serum HDL < 40 mg/dL for men and < 50 mg/dL for women; serum TC ≥ 240 for men and women; and serum TG ≥ 150 mg/dL for men and women [29]. The calculated ratio of LDL/HDL levels > 2.5 was also considered as high [30].

### Anthropometric measurement

Height and body weight were measured by a trained investigator. Height was measured using a wall-fixed tape measure without bumps with an accuracy of 0.1 cm. Moreover, body weight was measured while the participants were in light clothing and without shoes to the nearest 100 g using a digital scale (SECA, model 755, Germany). The body mass index (BMI) was computed by dividing weight (kg) by height (meters) squared.

### Physical activity measurement

Physical activity information was collected using the international Physical Activity Questionnaire (IPAQ) through face-to-face interviews every week, then it converted to the metabolic equivalent per week (MET-h/wk) [31] and classified to sedentary, moderate, and active based on the median of the MET-h/week levels.

### Assessment of other variables

Data on other variables including age, gender, marital status (single, married, widowed, or divorced), BMI, smoking (yes/no), education (illiterate, primary school, middle and high school (diploma), university or college degree and postgraduate (university)) were obtained from the questionnaires. Likewise, economic status was collected using a predefined questionnaire in which the interviewers asked about house ownership, area of the house, number of bedrooms, house amenities (whether they have a freezer, washing machine, dishwasher, computer, bathroom, color television, vacuum cleaner, mobile phone, laptop, internet access, etc.) whether they travel within the country or had any foreign pilgrim trips and the number and type of car that each participant owns. Each item was given a specific score, and the participants of the study were categorized into low, medium, and high economic status based on tertiles of the overall summed economic status score.

### Statistical methods

The scores of the DASH/MED diet were categorized into tertiles. Values for continuous variables were presented as mean and their standard deviation (SD). Analysis of variance and the chi-square test were performed for continuous and categorical variables respectively, to compare the general characteristics of participants across categories of DASH/MED scores. Binary logistic regression was fitted in several models to assess the associations between tertiles of DASH/MED scores and lipid profiles. Besides, analysis of covariance (ANCOVA) was used for adjustment of potential confounders comparing lipid profiles between categories of DASH/MED scores. Age (year), sex (male/female), and energy intake (kcal/day) were adjusted in the first model. Moreover, in the second model, additional adjustments were done for physical activity (inactive/moderate/active), education (illiterate/primary school/diploma/university), marital status (married/single/divorced/widow), BMI, smoking (yes/no), and socioeconomic status (high/moderate/ low). In all these analyses, the first tertile of DASH/MED score was considered as a reference. By using multivariable logistic regression analysis, the odds ratio (OR) with a corresponding 95 % confidence interval (CI) were calculated to quantify the association of lipid profiles with DASH/MED diet scores. The odds ratios’ trend across increasing categories of DASH/MED diet, was assessed using the median score in each category as a continuous variable. The adherence to the MED diet score was defined as high if the score was ≥ 6 points (the third tertile), medium if the score was 3–6 (the second tertile), and low if the score was ≤ 3 points (the first tertile). The same was done for the DASH diet score, but the cut-off was ≥ 51 for the high, or 41–50 for the medium, or ≤ 40 for the low adherence category. All statistical analyses were conducted using a statistical package for social sciences (SPSS), version 20 (SPSS Inc., Chicago, III, USA). *P* ≤ 0.05 was considered statistically significant.

## Results

**Fig. 1:**
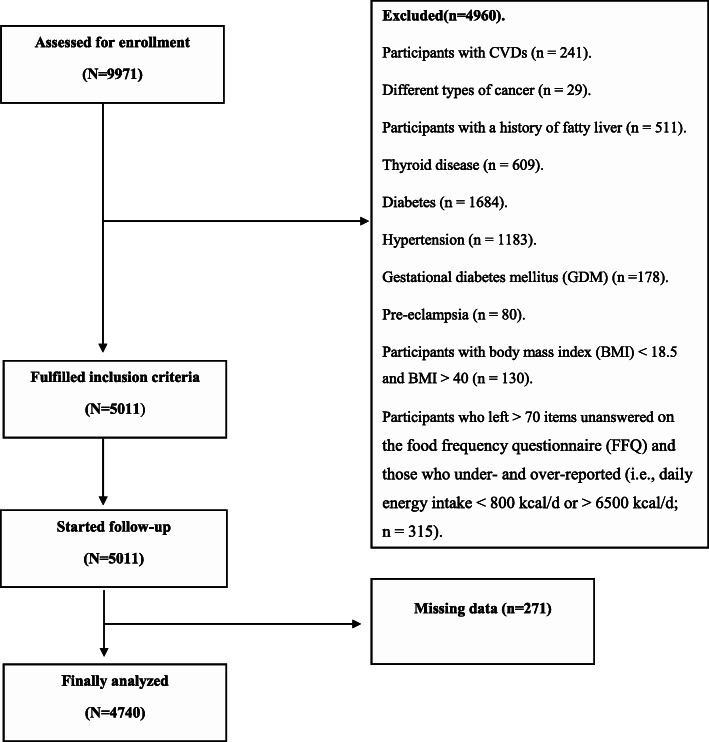
Study flowchart

Overall, 4740 participants (2856 men vs. 1884 women), (mean age and SD: 50 ± 8.6 years) remained and were included in the current analysis (Fig. 1). General characteristics of study participants according to tertiles of DASH and MED dietary patterns are presented in Table 1. Subjects with a high score of MED diet were more likely to be men and married. Besides, those with a high score of the DASH diet were older, more likely to be men, and had a smoking history. There were no other significant differences in general characteristics between tertiles of the DASH/MED score.

**Table 1:** Characteristics of study participants according to tertiles of DASH and MED dietary patterns^1^

	MED				DASH			
	T1 (***n*** = 1771)	T2 (***n*** = 1181)	T3 (***n*** = 1788)	***P***-value^**2**^	T1 (***n*** = 1475)	T2 (***n*** = 1840)	T3 (***n*** = 1425)	***P***-value^**2**^
**Sex**				0.01^*^				0.01^*^
Male	62.3 (1104)	61.1 (722)	57.6 (1030)		62.6 (923)	57 (1049)	62 (884)	
Female	37.7 (667)	38.9 (459)	42.4 (758)		37.4 (552)	43 (791)	38 (541)	
**Age (year)**				0.10				0.01^*^
30-39	7.1 (126)	6.8 (80)	6.5 (116)		7.4 (109)	7.5 (138)	5.3 (75)	
40-49	48.2 (854)	48.6 (574)	45.6 (815)		54.8 (809)	46.4 (853)	40.8 (581)	
50-59	29.5 (522)	29.2 (345)	31 (555)		26 (384)	30.5 (561)	33.5 (477)	
≥ 60	15.2 (269)	15.4 (182)	16.9 (302)		11.7 (173)	15.7 (288)	20.5 (292)	
**BMI**				0.36				0.42
**Normal**	28.2 (501)	33.0 (390)	27.9 (500)		30.5 (450)	31.0 (590)	28.0 (500)	
**Overweight**	33.5 (594)	25.4 (301)	32.2 (630)		33.4 (494)	31.5 (600)	35.7 (510)	
**Obese**	31.1 (552)	41.0 (485)	29.6 (531)		27.1 (401)	26.6 (506)	39.2 (559)	
**Married status**				0.03^*^				0.80
Single	25.9 (458)	25.1 (297)	25.2 (450)		24.4 (360)	26.1 (480)	25.6 (365)	
Married	62.1 (1099)	59.4 (702)	60.9 (1089)		62.4 (921)	59.8 (1100)	61 (869)	
Divorced	11.3 (208)	14.8 (175)	13.3 (237)		12.8 (185)	13.7 (253)	12.8 (183)	
**Education**				0.08				0.26
Illiterate	29.4 (521)	29 (343)	28.7 (513)		27.1 (399)	29.9 (550)	30 (428)	
Primary school	19.7 (349)	19.9 (235)	19.1 (342)		20.7 (306)	19.2 (354)	18.7 (266)	
Diploma^3^	23 (408)	24.8 (293)	22.6 (404)		25 (369)	22.8 (419)	22.2 (317)	
University	26.9 (393)	26.2 (310)	29.7 (527)		19.9 (280)	18.3 (316)	29.1 (414)	
**Smoking**				0.33				0.01^*^
No	73 (1292)	72.1 (852)	73.9 (1321)		75 (1028)	74.4 (1368)	69.7 (1069)	
Yes	25.3 (448)	26.3 (311)	23.8 (425)		28.9 (427)	23.6 (435)	22.6 (322)	
**Physical activity**				0.69				0.40
Sedentary	39.2 (502)	29.8 (564)	31.8 (446)		27.3 (380)	36.3 (663)	35.5 (507)	
Moderate	33.5 (494)	34.3 (613)	32.9 (472)		33.5 (494)	33.3 (613)	33.1 (446)	
Active	27.3 (380)	36.3 (663)	35.5 (507)		39.2 (502)	29.8 (564)	31.8 (446)	
**Social-economic status**				0.28				0.36
Poor	25.8 (381)	26.9 (494)	33.5 (478)		33.5 (478)	28.8 (381)	26.9 (494)	
Moderate	33.4 (492)	37 (683)	32.3 (460)		32.3 (460)	33.4 (492)	37 (683)	
Good	37.4 (552)	36.3 (663)	34.2 (487)		34.2 (487)	37.4 (552)	36.3 (663)	
**Total energy intake**				0.16				0.27
800-2225	28.2 (500)	28.9 (342)	22.5 (403)		24.3 (359)	24.6 (453)	30.1 (430)	
2225-3650	18.2 (324)	19 (225)	22.7 (406)		23.7 (350)	19.9 (367)	25.2 (360)	
3650-5075	22.7 (403)	26.2 (310)	22.2 (398)		30.7 (453)	25.4 (468)	22.4 (320)	
5075-6500	18.2 (490)	21.6 (256)	23.8 (427)		21 (310)	22.9 (423)	21.5 (307)	

^1^Data are reported as a percent (number). T1: first tertile; T2: second tertile; T3: third tertile

^2^Chi-squared test

^3^Diploma: middle school diploma, and high school diploma

^*^Values with distinct superscripts are significantly different at *P* < 0.05

The mean and standard deviation of variables is indicated in Table 2. Participants with high adherence to the MED diet had a higher level of serum HDL (52.8 ± 12.3 vs. 51.6 ± 11.6; *p*-value: 0.01; third vs. the first tertile). Other blood lipid markers were not improved by the MED diet. Furthermore, individuals with high adherence to DASH diet in comparison with the first category had a lower level of serum TC (189.9 ± 40.1 vs. 191.9 ± 41.2; *p*-value: 0.01), TG (158.6 ± 90.8 vs. 167.4 ± 104.4; *p*-value: 0.01), LDL (106.7 ± 32.0 vs. 108.3 ± 31.8; *p*-value: 0.01) and a low ratio of LDL/HDL (2.10 ± 0.7 vs. 2.17 ± 0.7; *p*-value: 0.01). However, the adjustment of the confounder variable could not demonstrate the high level of serum HDL (52.5 ± 12.3 vs. 51.3 ± 11.6; *p*-value: 0.06).

**Table 2:** Mean and standard deviation (SD) for lipid profile across tertiles of DASH and MED dietary patterns^1^

	MED				DASH					
	T1	T2	T3	***P***-value^**2**^	***P***-value^**3**^	T1	T2	T3	***P***-value^**2**^	***P***-value^**3**^
**TC** ^**4**^	190.4 ± 39.1	190.7 ± 44.9	191.3 ± 38.7	0.81	0.78	191.9 ± 41.2	190.7 ± 40.1	189.9 ± 40.1	0.41	0.01^*^
**TG** ^**4**^	161.3 ± 98.8	163.3 ± 95.0	159.2 ± 95.2	0.53	0.89	167.4 ± 104.4	157.7 ± 94.3	158.6 ± 90.8	0.01^*^	0.01^*^
**HDL** ^**4**^	51.6 ± 11.6	52.10 ± 12.2	52.8 ± 12. 3	0.01^*^	0.04^*^	51.3 ± 11.6	52.6 ± 12.1	52.5 ± 12.3	0.01^*^	0.06
**LDL** ^**4**^	107.7 ± 31.2	106.5 ± 31.6	107.7 ± 30.6	0.54	0.78	108.3 ± 31.8	107.3 ± 29.9	106.7 ± 32.0	0.42	0.01^*^
**LDL/HDL** ^**4**^	2.16 ± 0.7	2.12 ± 0.7	2.12 ± 0.7	0.16	0.23	2.17 ± 0.7	2.12 ± 0.7	2.10 ± 0.7	0.03^*^	0.01^*^

^1^Values are reported as mean ± SD. T1: first tertile; T2: second tertile; T3: third tertile

^2^one-way ANOVA

^3^Adjusted for age, sex, and energy intake (Kcal/day), marital status (married/single/divorced or widowed), BMI, physical activity (sedentary/moderate/active), education level (illiterate/ primary school/ high school diploma/college and university), smoking (no/ yes), economic status (poor/ moderate/ good) using analysis of covariance (ANCOVA)

^4^*TC* total cholesterol, *TG* triglyceride, *HDL* high-density lipoprotein, *LDL* low-density lipoprotein

^*^Values with distinct superscripts are significantly different at *P* < 0.05

Table 3 provided a multivariable-adjusted odds ratio for lipid profiles across tertiles of the DASH/MED diet. Greater adherence to MED diet was directly associated with 15 % lower odds of high LDL/HDL ratio [(OR: 0.85; 95 % CI: 0.73–0.99); third vs. the first tertil]. Also, after controlling for all potential confounders, we found a significant relationship between the MED diet and the LDL/HDL ratio [OR: 0.85; 95 % CI: 0.72–0.99], third tier vs. first tier. Besides, we observed a decreasing trend with higher adherence to the MED diet in crude and adjustment of all confounder variables (*p* for trend = 0.04).

**Table 3:** Multivariate adjusted odds ratios and 95 % confidence intervals for lipid profile based on tertiles of DASH and MED dietary patterns^1^

	MED				DASH			
	T1	T2	T3	***P***-trend	T1	T2	T3	***P***-trend
**High TC** ^**2**^								
Crude	1	0.90 (0.77 – 1.05)	1.06 (0.92 – 1.21)	0.38	1	0.91 (0.79 – 1.05)	0.95 (0.81 – 1.05)	0.51
Model 1^†^	1	1.06 (0.77 – 1.05)	1.03 (0.90 – 1.19)	0.60	1	0.86 (0.75 – 1.00)	0.83 (0.71 – 0.97)^*^	0.02^*^
Model 2^‡^	1	0.88 (0.75 – 1.03)	1.01 (0.87 – 1.16)	0.89	1	0.86 (0.84 – 0.99)^*^	0.81 (0.69 – 0.95)^*^	0.01^*^
**High TG** ^**2**^								
Crude	1	1.07 (0.92 – 1.24)	0.96 (0.84 – 1.10)	0.65	1	0.88 (0.77 – 1.02)	0.92 (0.79 – 1.06)	0.27
Model 1^†^	1	1.09 (0.94 – 1.26)	0.99 (0.87 – 1.14)	0.97	1	0.92 (0.80 – 1.06)	0.90 (0.77 – 1.05)	0.19
Model 2^‡^	1	1.09 (0.93 – 1.27)	1.01 (0.88 – 1.16)	0.86	1	0.92 (0.80 – 1.06)	0.99 (0.77 – 1.05)	0.19
**Low HDL** ^**2**^								
Crude	1	0.96 (0.82 – 1.14)	1.05 (0.90 – 1.23)	0.48	1	1.08 (0.92 – 1.26)	1.08 (0.91 – 1.27)	0.36
Model 1^†^	1	0.96 (0.81 – 1.14)	1.06 (0.91 – 1.24)	0.41	1	1.07 (0.91 – 1.26)	1.03 (0.87 – 1.23)	0.64
Model 2^‡^	1	0.97 (0.81 – 1.15)	1.03 (0.88 – 1.21)	0.65	1	1.07 (0.91 – 1.26)	1.01 (0.85 – 1.20)	0.84
**High LDL** ^**2**^								
Crude	1	0.84 (0.70 – 1.01)	0.97 (0.81 – 1.13)	0.72	1	0.85 (0.71 – 1.00)	0.94 (0.79 – 1.12)	0.52
Model 1^†^	1	0.84 (0.69 – 1.01)	0.95 (0.81 – 1.11)	0.55	1	0.80 (0.68 – 0.95)^*^	0.84 (0.70 – 1.01)	0.07
Model 2^‡^	1	0.84 (0.70 – 1.02)	0.93 (0.79 – 1.10)	0.46	1	0.80 (0.67 – 1.96)	0.83 (0.69 – 1.00)	0.05
**LDL/HDL** ^**2**^								
Crude	1	0.92 (0.78 – 1.08)	0.85 (0.73 – 0.99)	0.04^*^	1	0.82 (0.66 – 0.91)^*^	0.82 (0.70 – 0.95)^*^	0.01
Model 1^†^	1	0.92 (0.78 – 1.09)	0.87 (0.74 – 1.01)	0.07	1	0.72 (0.61 – 0.86)^*^	0.82 (0.70 – 0.97)^*^	0.01
Model 2^‡^	1	0.91 (0.77 – 1.08)	0.85 (0.72 – 0.99)^*^	0.04^*^	1	0.72 (0.60 – 0.85)^*^	0.82 (0.70 – 0.96)^*^	0.01

^1^Values are reported as odds ratio and 95 % confidence interval. T1: first tertile; T2: second tertile; T3: third tertile

^2^*TC* total cholesterol, *TG* triglyceride, *HDL* high-density lipoprotein, *LDL* low-density lipoprotein

^†^Adjusted for age, sex, and energy intake (Kcal/day)

^‡^Adjusted for marital status (married/single/divorce or widowed), BMI, physical activity (sedentary/moderate/active), education level (illiterate/ primary school/ high school diploma/college and university), smoking (no/ yes), economic status (poor/ moderate/ good) plus variables in model 1

^*^Values with distinct superscripts are significantly different at *P* < 0.05

After adjustment for potential confounder variables (age, sex, energy intake), participants in the third tertile of the DASH diet had 17 % lower odds of high serum TC level in comparison with those in the first tertile (OR: 0.83; 95 % CI: 0.71–0.97). likewise, Additional adjustment based on marital status, BMI, physical activity, education level, smoking, and social-economic status result in a 19 % lower risk of having a high TC level [(OR: 0.81; 95 % CI: 0.69–0.95); third vs. the first tertile)]. Moreover, a decreasing trend with a higher tendency to the DASH diet after full adjustments for all potential confounders was observed (p for trend: 0.01). Furthermore, we observed an 18 % decrease in odds of the ratio of LDL/HDL in those with high adherence to the DASH diet [(OR: 0.82; % CI: 0.70 – 0.96); third vs. the first tertile].

## Discussion

Our findings from the current large-scale observational study suggest that the top category of the DASH diet might be significantly associated with lower serum levels of TC, TG, LDL, and the LDL/HDL ratio. Besides, high adherence to the MED diet could be directly related to an increase in the HDL level after controlling for potential confounders. Also, adjustment of confounding variables revealed a lower odd of LDL/HDL ratio in the third tertile of the MED diet. Moreover, high adherence to the DASH diet (third tertile), lower odds of high serum TC level and high LDL/HDL ratio were observed.

Numerous studies have reported an association between the MED diet and the risk of CVDs [32–34]. The more people adhere to MED dietary pattern, the less they have CVD risk factors [32]. It is shown that the protective effect of the MED pattern in individuals with history of infarction can be continued up to 4 years after the first infarction [35]. However, few have focused specifically on lipid profile as a primary outcome. Also, an updated meta-analysis that included 12 studies and 1,574,299 participants indicated that adherence to a MED diet was associated with a significantly lowered risk of mortality from cardiovascular diseases [36]. Previous studies have suggested that adherence to the MED diet might be associated with a lower risk of high TC [37]. Besides, in agreement with our results, another study found high HDL levels with adherence to the MED diet [38].

A publication on the Nurses’ Health Study cohort (NHS) with a follow-up of 24 years suggests that adherence to a DASH diet might contribute to a lower risk of coronary heart disease and stroke in middle-aged women [28]. Also, consistent with our results, the DASH diet has been shown to be effective in lowering plasma TC level and LDL/HDL ratio [39]. It is believed that the DASH diet has a favorable effect on lipid profiles because of its beneficial components. Higher amounts of fruits and vegetables in the DASH diet which increase the content of dietary fiber and phytoestrogens might respond to its beneficial effects on serum TG, total cholesterol, and LDL levels [39–41]. Also, the DASH diet contains higher amounts of non-hydrogenated vegetable oils that might contribute to favorable effects on lipid profiles. Several studies suggest that consuming edible vegetable oils has a modulating effect on blood pressure and serum lipid profiles [42, 43]. Similar beneficial effects on serum levels of TC, TG, LDL, and LDL/HDL ratio were found in our study as well.

It should be noted that we observed some differences between the effects of the MED diet and the DASH diet on blood lipids. In the present study, the MED diet tended to be directly associated with a high HDL level, simultaneously with lower odds of LDL/HDL ratio, while there was no significant association between MED and other lipid profiles; Nevertheless, the DASH diet performed somewhat better than the MED, having significant inverse associations with high TC, TG, LDL levels, and the LDL/HDL ratio. The difference in the study region between the current study and previous studies may be one of the main reasons why such results were reported. The MED dietary pattern in Mediterranean countries differs from other countries such as Iran [44]. Although the most consumed cereals in Iran belong to white rice and refined grains [45], the majority of people in the Mediterranean region consume brown rice and whole grains [44]. Furthermore, the amount of fish consumed and the methods used to prepare it vary across these regions, resulting in variations in omega-3 intake. For instance, in Mediterranean countries, olive oil is consumed as a main component of the diet and also to prepare fish. The nutritional value of fish may increase due to increased intake of antioxidants, phenols, and vitamins from olive oil consumption [44]. Nevertheless, Iranian people use corn oil and sunflower oil for the preparation of fish and other foods, which contain less proportion of unsaturated fatty acids, especially oleic acid than olive oil [46].

An important question that arises concerning our findings is whether the non-protective effect of the MED diet is reliable, due to lower consumption of whole-grain foods (< 10 g/day) and more consumption of refined grains (such as rice and white bread) in Iranian population that might affect our findings [47]. Moreover, we also attempted to take lifestyle into account by adjusting all analyses for physical activity and smoking. However, the odds result from the adjusted and unadjusted models were quite similar. This finding suggests that we identified an effect of diet, rather than other factors, on the odds of lipid profile, although we cannot rule out residual confounding because of the suboptimal measurement of these factors.

This study has several limitations as follows: First, our study was a cross-sectional study; thus, determining causal relationships between observed findings and lipid profile is not possible. We tried to minimize the potential confounding effects by excluding individuals with CVDs, type 2 diabetes, hypertension, cancer, and other chronic diseases since their serum blood lipids and also their dietary pattern might be affected. Likewise, participants who may have changed their eating habits due to illness were also excluded. Second, we used semi-quantitative FFQs to collect dietary assessment data with trained interviewers. Although using a validated questionnaire, the nature of FFQ is likely to cause misclassification. Third, the study was conducted on selected participants from Yazd Greater Area. Therefore, the generalization of our findings to the whole Iranian population should be done with caution. Moreover, although we tried to control the maximum number of potential confounding variables, residual confounding from unknown or unmeasured confounders cannot be excluded. On the other hand, the present study benefited from a large sample size, which might be its key strength. The study also exploited the information available on several non-dietary variables, allowing us to control for their supposed confounding effect in the analyses.

## Conclusions

The current large-scale cross-sectional study suggests a positive association between the DASH diet and serum levels of TC, TG, LDL, and LDL/HDL ratio, whereas the MED diet could increase the HDL and decrease LDL/HDL ratio. Further large-scale prospective studies are highly recommended to confirm our findings.

## Data Availability

The datasets used and/or analyzed during the current study are available from the corresponding author on reasonable request.

## Abbreviations

DASH: Dietary approach to stop hypertension
MED: Mediterranean diet
LDL: Low-density lipoprotein
HDL: High-density lipoprotein
TC: Total cholesterol
TG: Triglyceride
FFQ: Food frequency questionnaire
CVDs: Cardiovascular Diseases
FCT: Food composition table.
SSB: Sugar-sweetened beverage
BMI: Body mass index
OR: Odds ratio
SD: Standard deviation
CI: Confidence interval
